# Event and Comment

**Published:** 1905-05-15

**Authors:** 


### THE DENTAL REGISTER.

Vol. LIX. May 15, 1905. No. 5.

### EVENT AND COMMENT.

### The Passing of the Hand Excavator.

Agents of supply houses carry burs by the peck, which means that burs are in great demand. In quantity, quality, size, form and finish they more than meet the requirements of the purchaser; but who except the oldest of operative dentists has ever heard the virtues of the hand excavator extolled by these everywhere-present and always-busy men. Time was when these most useful of cutting instruments were to be seen in depots and on the road by the thousands, and their good points were published to the dental world as the end of the law in steel. Now, however, if one would have a cutter of this class he must ask for it, and great is his disappointment on learning from inspection and inquiry that forms once indispensable and easily had have passed even from the memories of the men who designed, made and marketed them. There was a time when excavators did it all; to-day they merely supplement the bur.

And this suggests that the inordinate and unnecessary use of the bur is responsible for most of the torture complained of by tha long-suffering occupants of the chair, and the grudge they cherish against the dental engine. Few of our patients hesitate in their answer as to which they prefer, the machine-operated bur or the hand-manipulated excavator. Without a doubt we use a bur too much; we use it when and where it is not only unnecessary but undesirable, whether from the view-point of the patient or that of the operator. It fails utterly in many cases where the hand-excavator measures up fully to the necessities of the case. Why not revive interest in and rehabilitate this most useful of accessories? Further delay may relegate the instrument and skill in its use to the list of arts that have been lost.—March *Office and Laboratory*.

As the old negro said when he first heard the President's emancipation proclamation, "dat am mighty good hearin."

There is no question but that we are getting careless in some respects of our patient's feelings in these days of electric engines and cheap burs. If every operator should go about once a month to a dentist who uses an electric engine with a flexible cable, and a hand-piece that is worn and sadly in need of a lubricant, and have a real sensitive tooth ground with a dull bur run at lightning speed, or even a filling polished with a corundum stone that has lost its original symmetry, if it ever had one, and he will conclude that a hand-excavator or a chisel should be used more frequently. The heroic work on the teeth can be done more expeditiously with the chisel and much more comfortably for the patient. The engine should do its best work in the formation of retention, and should be handled as delicately as a drawing pen. There is no question but that the modern electrical engine is a great aid to rapid work, but when this exhausts the patient and creates a dread of dental operations in the minds of our patients, it would seem after all that it would be more profitable to suffer some personal fatigue than to exhaust the vitality of our patients. It is so easy with a beautifully-running electric engine and a sharp bur to cut the tooth unnecessarily, that undoubtedly we many times exceed the necessities for conservative operations. Another danger that is new is the excessive heating of live teeth which have been anesthetized by the injection method of obtunding dentin. It must be an unusually-vigorous pulp that will withstand being forcibly infiltrated with a ten percent solution of cocain, and while comatose to be subjected to a physical influence which is unusual and likely to deprive it of its last resource for recovery. The delicate nerve cells tightly packed in an unyielding pulp chamber must be subjected to a disintegrating pressure from the molecular expansion of the excessive heat that endangers their recuperative powers. If we must use these helpful appliances, let us see that they are of the best kind, and always kept in prime condition, or as near that as is practicable. Above all they should be used with great discretion, as well as skill.

### Nominating State Board Officers.

Various State dental societies have had enacted into their dental laws a clause making it obligatory for the Governor to appoint members to serve on the State Examining Board from nominations made by the State Dental Society. Insisting on such a provision in the proposed new law for Illinois last year resulted in defeating the effort to get a reconsideration of the bill, as the Governor strenuously opposed the bill on that account. The supposition here was that the Governor was not willing to give up his opportunity of distributing his favors to whom he pleased for political rewards. Word now comes from Oregon that a bill has been introduced in the Senate of that State which takes away from the State Association its perogative of nominating candidates for such appointment. The reason given is that this matter has been the source of so much trouble in the association that it has nearly ruined it, and caused no inconsiderable trouble to the profession. The trouble seems to result from the fact that the nominations are made by the executive committee of the society instead of by the ballot of the whole society. It is plain to be seen that this method would promote trouble of the kind suggested, which would not occur if the whole association by secret ballot elected the three candidates to be recommended to the Governor from which to select his appointee· The idea is a good one, but the method of selecting the candidate is where the trouble comes in this case.

### Has the National Board of Examiners Power?

Dr. Charles R. E. Koch, in the March *Northwestern Dental Journal*, has an article on the "Evolution of Dental Education," in which he says the responsibility for fixing a standard of dental education rests with the National Association of Dental Faculties and the National Board of Examiners, and while both are voluntary associations they have no power to compel obedience. We presume he means to say neither have legal power, which is quite true. But it is a fact that either of these organizations has a *power* which is sufficient to force a compliance with any reasonable legislation they may choose to enact. Both organizations working together can establish a standard that no statutary enactment could more completely enforce. And since the National Board of Examiners represents the large majority of the influential States, it would seem that representing the people and the profession of th se States, it could not only with perfect propriety but legally say what should constitute a reputable and sufficient standard of education. If this body should emphatically declare itself on this question it would have a great influence in crystallizing public and professional sentiment into harmony with any reasonable standard. This in turn would influence the educational body to conform with the standard, and all future legislation would embody thi; as a minimum standard. Therefore we would suggest that all the power we need is embodied in this organization.

### Is the Advertising Dentist a Menace or a Necessity?

Ethical practitioners look upon the so-called advertising dentist as a quack and a nuisance, if not a professional menace. He is a quack of course when he publishes his purpose to do the impracticable and deceives the public by promising what he does not fulfill. He is a nuisance when he usurps the rights of honorable and qualified practitioners who build their business on public confidence rather than publicity and notoriety. It is possible that the advertising dentist may not be a quack in the strict definition of the term, but he is almost certain to interfere with other practitioners who are trying to conduct their practices on strictly ethical lines. He entices their patients away and educates the public to a fee basis that will not compensate the considerate practitioner. He must work for small fees, consequently he is compelled to cheapen the quality of his service, in order that the quantity may be large enough to bring the necessary financial returns.

And when this happens he not only becomes a professional nuisance, but a public menace. But some one will say that there are so many people that can not afford good service that they should have the opportunity to get what they can pay for rather than have none at all. The argument is good and also bad. The best dental service is none too good, and cheap service is often worse than none at all. Besides this it is the common boast of the advertising man that he gets as good fees as the majority of the strictly ethical, which is only one of the strongest proofs of the fact that these men are a public menace. The fact is that they get fees largely out of proportion to the value of the service rendered. The practice is carried on as a shrewd business venture and there is no such a thing as confidential relations. It may be true of course that there are exceptions to this statement and doubtless much good work is done in these offices, but the exception is not aimed at by those who go into th s kind of business. The effort is to create and supply a demand as a business proposition. The very fact that the advertising practitioner invites his patients on a special basis puts him at a disadvantage, he must either make concessions or convey unmistakably the impression that he has done so, or the patient leaves his office dissatisfied never to return. Since there are not enough ethical practitioners to take care of all those who want dental services, the advertiser may be a necessity, because he induces many people to care for their teeth in a partial or inferior way it may be. If he would do what he agrees to do there is no question but what he would meet a great need in our large cities where there are thousands needing professional services who would never go to the office of an ethical practitioner. To that extent he may claim justification for his method of conducting his business.

**Temporary Licenses Abandoned.**

The California State Board of Examiners has had repealed the section in the State dental law which gave the Board authority to issue a temporary license to practice until a meeting of the Board for examination. The reasons assigned are that the privilege has been abused; some to whom temporary licenses were granted never came up for examination, while others refused to take the examination after getting the temporary license. It has developed in several cases that practitioners from other States have come to California and after getting started in business have taken the examination and failed to pass, creating a very unsatisfactory state of affairs, and giving the rejected candidate what he considers a good reason for the Board to make exceptions in his case. Then again the privilege is given to any member of the Board to issue a temporary license without consulting any other member of the Board. It has been an unpleasant experience and the Board is thankful to have this section repealed. We have often thought that if a person holding a temporary license to practice would go to the trouble of contesting the law, he would be able to register permanently because of his temporary license. If a dentist is qualified to practice six months, or even a day, is not that good evidence that he is capable of practicing as long as he wishes? It would not require much argument to make a strong provisional case on that point. Another very unwise provision of this feature in the California law was that the member granting the temporary license collected for his own use a fee of five dollars.

**California's Annual License Fee Constitutional.**

The California dental law has a clause which compels every licensed dentist in the State to pay each year to the State Dental Examining Board a fee of two dollars, which shall be used for prosecuting persons who attempt to practice dentistry illegally in the State, and also to cover the expense of collecting it. The Supreme Court of the State has recently affirmed the constitutionality of the law on this point especially. The Board will now take steps to collect the fees, as a large number have refused to pay. The method will be to send to each dentist, who does not promptly send in his two dollars, a registered letter notifying him that his annual license fee is due, and if not paid within thirty days, his license will be annulled and his name taken from the books of the registered dentists of the State. The wise man will, of course, comply with the law and pay promptly. It will be strange if many do not neglect or refuse to pay within the option and so be compelled to apply for reinstatement at an expense of the total uncollected fees and a fine of not to exceed ten dollars. Should one forfeit his registration, he will be compelled to take a new examination for registration. A dentist who allows his license to lapse in any way becomes an illegal practitioner, no matter how long he has practiced in the State, or how skillful he may be. It strikes us that this is a very good feature, and as soon as the profession becomes accustomed to paying this fee each year there will be no complaint, especially if the Board makes a proper use of the money.

